# Effects of computer-aided rowing exercise systems on improving muscle strength and function in older adults with mild knee osteoarthritis: a randomized controlled clinical trial

**DOI:** 10.1186/s12877-022-03498-2

**Published:** 2022-10-21

**Authors:** Pei-Ling Lin, Lee-Fen Yu, Shu-Fen Kuo, Xin-Miao Wang, Liang-Hsuan Lu, Chueh-Ho Lin

**Affiliations:** 1 Master Program in Long-Term Care, College of Nursing, Taipei Medical University, Taipei, Taiwan; 2 Department of Nursing, Taipei Medical University, Taipei, Taiwan; 3 Center for Nursing and Healthcare Research in Clinical Practice Application, Wan Fang Hospital, Taipei Medical University, 250 Wu-Xing Street, Taipei, 11031 Taiwan; 4 School of Nursing, College of Nursing, Taipei Medical University, Taipei, Taiwan; 5 Faculty of Humanities, Zhejiang Dong Fang Polytechnic College, Wenzhou, China; 6 Department of Physical Therapy and Assistive Technology, National Yang Ming Chiao Tung University, Taipei, Taiwan

**Keywords:** Older adults, Computer-aided system, Rowing exercise, WOMAC, Lower joint impact, Osteoarthritis

## Abstract

**Background:**

Osteoarthritis (OA) is common in aged adults and can result in muscle weakness and function limitations in lower limbs. Knee OA affects the quality of life in the elderly. Technology-supported feedback to achieve lower impact on knee joints and individualized exercise could benefit elderly patients with knee OA. Herein, a computer-aided feedback rowing exercise system is proposed, and its effects on improving muscle strength, health conditions, and knee functions of older adults with mild knee OA were investigated.

**Methods:**

Thirty-eight older adults with mild knee OA and satisfying the American College of Rheumatology (ACR) clinical criteria participated in this randomized controlled clinical trial. Each subject was randomly assigned to a computer-aided rowing exercise (CRE) group (*n* = 20) or a control group (CON) (*n* = 18) that received regular resistance exercise programs two times per week for 12 weeks. Outcome measurements, including the Western Ontario and MacMaster Universities (WOMAC), muscle strength and functional fitness of the lower limbs, were evaluated before and after the intervention.

**Results:**

Participants’ functional fitness in the CRE group exhibited significantly higher adjusted mean post-tests scores, including the WOMAC (*p* = 0.006), hip abductors strength (kg) (MD = 2.36 [1.28, 3.44], *p* = 5.67 × 10^–5^), hip adductors strength (MD = 3.04 [1.38, 4.69], *p* = 0.001), hip flexors strength (MD = 4.01 [2.24, 5.78], *p* = 6.46 × 10^−5^), hip extensors strength (MD = 2.88 [1.64, 4.12], *p* = 4.43 × 10^−5^), knee flexors strength (MD = 2.03 [0.66, 3.41], *p* = 0.005), knee extensors strength (MD = 1.80 [0.65, 2.94], *p* = 0.003), and functional-reach (cm) (MD = 3.74 [0.68, 6.80], *p* = 0.018), with large effect sizes (η^2^ = 0.17–0.42), than those in the CON group after the intervention.

**Conclusions:**

Older adults with knee OA in the CRE group exhibited superior muscle strength, health conditions, and functional fitness improvements after the 12-week computer-aided rowing exercise program than those receiving the conventional exercise approach.

**Trial registration:**

The Institutional Review Board of the Taipei Medical University approved the study protocol (no. N201908020, 27/05/2020) and retrospectively registered at ClinicalTrials.gov (trial registry no. NCT04919486, 09/06/2021).

## Background

Osteoarthritis (OA) is a common disorder in elderly people and can result in the progressive deterioration of the structural integrity of joints. Pereira et al. reported that OA is prevalent in approximately 10–20% elderly population [1, 2]. Furthermore, they revealed that more than four-fifths of OA cases occur mainly in the knee joints [3]. The clinical symptoms for OA in knee joints include joint stiffness, pain, and functional disabilities, which may decrease the quality of life (QOL) for people with OA [4]. Jessica et al. revealed that the medical cost of treating patients with OA is $460 billion, and it is increasing annually [2], which may result in a subsequent rise in the care and economic burden of families in an aging society [5, 6]. Therefore, the development of appropriate exercise devices and measures to ameliorate muscle weakness and functional disabilities in older adults with knee OA and improve their QOL have become a critical topic of research.

Many clinical approaches, including surgery, medication, and exercise have been proposed to alleviate the symptoms of patients with knee OA. However, medication results in many side effects [7]. With increasing age, the risks and complications from surgery increase considerably [8]. By contrast, an early study reported that exercise is a safe clinical approach to reduce pain and improve function [9–11]. Examination of the European Society for Clinical and Economic Aspects of Osteoporosis, Osteoarthritis and Musculoskeletal Diseases, and Osteoarthritis Research Society International guidelines revealed that exercise is a crucial treatment for people with OA [12]. Studies have indicated that exercises (aerobic and strengthening exercises) can considerably improve muscle strength and function in clinical approaches [12–14]. However, many knee OA patients reported they feeling uncomfortable and pain in their OA knee joints when performing aerobic and strengthening exercises, such as straight-leg raise, step-ups, squatting, biking, and low-speed running, in the clinic [15, 16]. Therefore, the development of an appropriate exercise with low joint impacts and loading of OA knee joints is critical for improving the muscle strength and function in the lower limbs of older adults with knee OA. The results of numerous studies are consistent with these findings [17, 18].

Recent studies reported that Tai Chi exercises are safe and slow movement multicomponent exercises with low impacts on the OA knee joint in older adults [18, 19], and they improve physical performance, functional fitness, and psychological health conditions in older adults [18, 20–23]. Although Tai Chi can significantly improve physical performance and reduce pain and stiffness in individuals with OA [18, 22, 23], several limitations were also reported, including the requirement of well-experienced Tai Chi exercise instructor, inadequate feedback in group exercises, being insufficiently challenging, and high withdrawal rate because of boredom [18, 24]. By contrast, combining game-based feedback and exercise can considerably enhance motivation, muscle strength, range of motion, and adhesion for people with knee disorders [25–29]. Flexible computer-aided exercise program development can provide custom feedback and quantitative exercise training in the clinic. Therefore, this study developed a computer-aided feedback and low weight-bearing rowing exercise system by providing custom challenge setting and game-based feedback. Furthermore, the study investigated the effect of the proposed method on improving the muscle strength and function in the lower limbs of older adults with mild knee OA. We hypothesized that computer-aided rowing exercises can improve the muscle strength, health conditions, and function of older adults with knee OA.

## Methods

### Study design

**Fig. 1:**
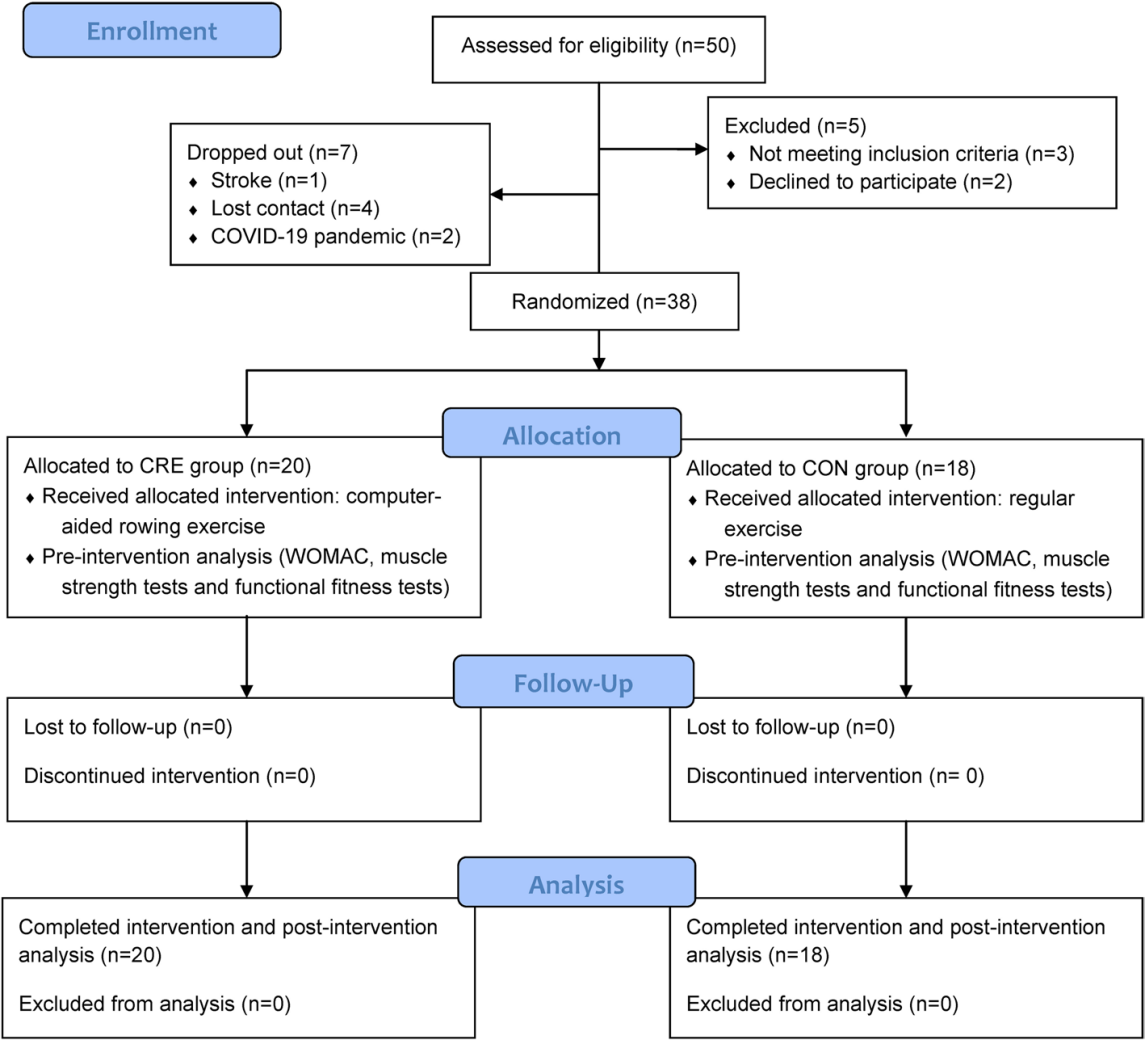
Flow diagram of the randomization procedure and outcome measurements of the study

This study was a single-blinded (the outcome evaluator was blinded when assessing data) intervention study. Randomized control trial comparing computer-aided rowing exercise (the experimental group, CRE) and regular exercise programs (control group, CON) were compared (trial registry no. NCT04919486) to investigate the effect of computer-aided rowing exercises on WOMAC, muscle strength, and functions of the knee OA joints in elderly adults (Fig. 1). We enlisted the services of a research assistant for ensuring that all participants adhered to the computer-aided rowing and regular exercise programs in both groups during the study period. The statistician analyzing the functional fitness data was also blinded.

### Participants

The sample size calculation was performed based on the muscle strength findings in lower limbs on OA patients [30] via G*power using a medium effect size of 0.31, *ɑ* of 0.05, and power of 0.95. A minimum sample size of 36 participants was required. Furthermore, regarding the 10% dropout rate, 40 participants were enrolled for this study. Forty-five older adults with knee OA were identified by physicians using the clinical criteria of the American College of Rheumatology (ACR) [31]. All subjects were orally recruited by researcher from communities in the study and following the inclusion and exclusion criteria between October 2020 and November 2020 (this study was approved and valid from September 1, 2019 to June 30, 2021). Inclusion criteria were as follows: 1) age 65–85 years, 2) unilateral knee OA and meeting the ACR criteria for mild knee OA [31], 3) mini-mental status examination score of ≥ 25 and fluency in Chinese [32]; 4) ability to perform tests without feeling uncomfortable; and 5) ability to stand up, sit down, and walk without using assistive devices. The exclusion criteria were: 1) surgery on the knee or hip joints; 2) lower limb fractures or severe medical condition or pain, and inability to perform exercises for knee OA; 3) the presence of acute inflammation in the past 6 months, 4) severe cardiovascular and heart failure; 5) neurological diseases affecting the motor and functions of the lower limbs; or 6) inability to understand and follow study procedures. However, a participant suffered a stroke, four participants lost contact, and two participants stated that they could not follow the protocols because of the COVID-19 pandemic. Thus, seven participants dropped out before the study began. Finally, 38 participants were considered in the study and were randomly assigned to the CRE (*n* = 20) and CON groups (*n* = 18). The investigator used a generated random number (1 = CRE, 2 = CON) for each subject for randomization based on LabVIEW (2015 edition, National Instruments, Austin, TX. USA). A flowchart of the randomization and outcome measurements is displayed in Fig. 1. Informed consent was obtained from all participants before the study. The institutional review board of Taipei Medical University approved the study protocol (no. N201908020).

### Instrumentation

#### System architecture of the computer-aided rowing exercise system

##### Hardware architecture

**Fig. 2:**
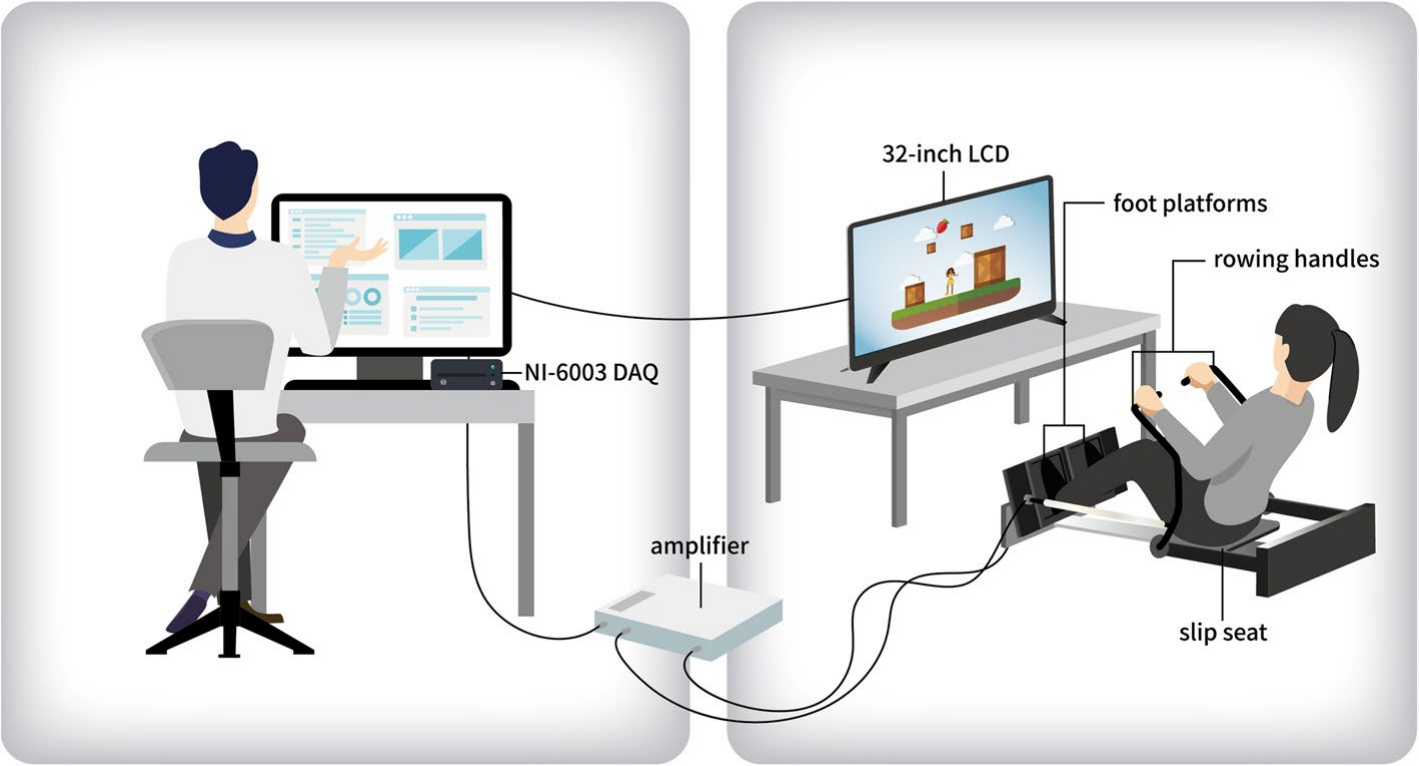
Participant performing the computer-aided rowing exercise in front of the liquid crystal display (LCD) monitor

To enable participants to perform rowing exercises that induce low weight bearing at the knee joints in the lower limbs, a CRE system was designed using Accu Balance Company (New Taipei City). The system was made of steel and included two foot platforms, two load cells (TEDEA 615 Huntleigh Co.), two rowing handles, a slip seat with spring (spring tension adjustment 22 kg, Accu Balance Co.), a signal amplifier, an NI-6003 data acquisition card (DAQ, 16-bit analog-to-digital converter, National Instruments Corp., Austin, TX, USA), a 32-inch LCD, and a laptop computer (Fig. 2). Each load cell was mounted under the foot platform and measured 980 N to record the leg press force of the lower limbs during the rowing exercise. The signals were amplified and sent to the DAQ card through a Bayonet Neill-Concelman (BNC) connector and then transmitted to a computer. Furthermore, two rowing handles were provided on the side to ensure stability in the upper body when performing the rowing exercise.

##### Software interface

LabVIEW 2015 (National Instruments Corporation, Austin, TX, USA) was used as the system software interface for storage, data analysis, and connecting the flash game. The sampling frequency was set at 1 kHz. The program interface of a CRE system includes: 1) a signal filtering module to filter signal noise; 2) a data management module was used to calculate the force data from load cells to obtain the leg press force during rowing exercise; and 3) a game control module to transmit the voltage data of both feet into the direction control (right and left) of the keyboard and then play the game. Furthermore, the target force values in the repetition maximum (RM) for triggering the direction control in the game for each subject should be entered into the interface. If the right leg generates a kick force higher than the target force value, it triggers the function of the right arrow key on the keyboard and vice versa for the left leg. Automatic calibration of this system was performed before the exercise for each subject.

#### Computer-aided rowing exercise program

The patients were seated comfortably on a slide seat with both feet on the foot platforms of the computer-aided rowing exercise system and placed their knees at 90° of flexion. Both hands grasped the sides of the seat. A 32-inch liquid crystal display (LCD) was placed in front of the subject and played a video game, which was projected and set on a computer. Before the intervention, the quadriceps 1-RM was determined and recorded according to a method listed previously [33]. Subsequently, the target force values in RM were entered at the interface. During training, patients fully extended the knee, kicked the foot platforms using a concentric quadriceps action, and subsequently flexed the knee using an eccentric quadriceps action. The target force for triggering the game was set at 50% of 1-RM during the first week and then progressively increased to 5% of the original 1-RM every 2 weeks [33, 34]. All subjects were trained 30-min in each section, with two sessions weekly for 12 weeks. During the exercise, subjects were asked to control the little man in the video game to push the box right or left and out of the plate, and each box was randomized and dropped from the top. Therefore, both lower extremities were trained.

#### Regular exercise program

During the study period, all participants in the CON group participated in regular exercise programs twice a week. Each session included 30 min of elastic band exercises (seated leg raises, standing leg side raises, leg press movement with elastic bands) guided by a physical therapist. Elastic bands were produced by THERA-BAND® (Hygenic Corp., Akron, OH, USA), and 60-cm red bands were selected for this study. A research assistant checked all participants without any other intervention. A certified blinded evaluator performed the WOMAC, muscle strength, and lower limb function assessments and collected data before and after the intervention. The research assistant also tracked the reasons for the number of missed sessions during the study period.

### Outcome measurements

The primary outcomes were the WOMAC score and muscle strength assessment. Functional fitness tests were used as the secondary outcomes. The Chinese version of the Western Ontario and MacMaster Universities (WOMAC) scale was used to evaluate the pain and function in this study [35]. The WOMAC is a well-established assessment tool [36, 37], and it has been widely used for evaluating pain, stiffness, and function in people with knee OA [35].

In this study, to assess the muscle strength in the affected lower limb, the MicroFET 3 hand-held dynamometer with excellent validity and reliability was used to evaluate the muscle strength in the lower limb [38], including the hip flexors, hip extensors, hip abductors, hip adductors, knee extensors, and knee flexors. The test procedure was performed according to the user manual and those of previous studies [38–40].

Functional fitness tests in lower limbs was evaluated to indicate the comprehensive abilities and assess the effects of the computer-aided rowing exercise system on the improvement of the functions related to ADLs in older adults with knee OA [41–45]. Functional fitness tests were performed before and after the 12-week intervention in both the CRE and CON groups, including the 30-s chair stand, functional reach, and 10-m walk tests.

### Statistical analysis

All data were pooled into a computer by the investigator and analyzed using the Statistical Package for the Social Sciences (SPSS) ver. 18.0 statistical software (SPSS, Chicago, IL, USA). First, the demographic data of the subjects were presented using descriptive statistics. The Shapiro–Wilk test was conducted to evaluate the normality of these data [46], and the independent sample t-test and Mann–Whitney U test of non-normality data were applied to analyze the differences in the WOMAC, muscle strength, and functional fitness values between groups at baseline. The chi-square test was performed for categorical data to compare the two groups. Cohens’ d of continuous data and Cramer’s V for categorical data were also applied for effect size between groups, where the value indicates small (0.2), medium (0.5), and large (0.8) effects [47, 48]. Second, the baseline data for the WOMAC score, muscle strength, and functional fitness revealed no heterogeneity between the CRE and CON groups. Paired sample t-tests and Wilcoxon signed rank test of non-normal data were performed to analyze the differences between the pre-and post-assessment within the two groups [49]. Cohens’ *d* was also applied for the effect size within the groups. Finally, we also performed the ANCOVA test and Quade’s ANCOVA test of non-normal data using an independent sample t-test to investigate the pre-and post-changes in the WOMAC, muscle strength, and functional fitness between groups [50]. The partial eta squared (*η*^2^) values of the ANCOVA tests and Quade’s ANCOVA test indicate effect sizes in accordance with Cohen’s guidelines (0.01, small; 0.06, medium; 0.14, large) [47]. Statistical significance was set at *p* < 0.05.

## Results

The normality of these continuous data was examined using the Shapiro–Wilk test and the results revealed the most normality distribution, in addition to the WOMAC and 10-m walk tests (s) (*p* = 0.013 and *p* < 0.001, respectively).

Table 1 presents the baseline characteristics of the participants’ backgrounds and functional fitness in the CRE and CON groups. All test results revealed that no significant differences occurred between the two groups, which indicated that CON exhibited superior hip abductors, hip adductors, hip flexors, hip extensors, and 10-m walk test results than the CRE group did.

**Table 1:** Baseline demographic data, muscle strength, and physical fitness values in the CRE and CON groups.

Variables	CRE group (*N* = 20)	CON group (*N* = 18)	95% Confidence interval		^*a*^*t/*^*b*^* X*^*2*^*/*^*c*^*Mann-Whitney U test*	*p*	Cohen’ *d/Cramer’s V*
			Lower	Upper			
Demographic data							
Age (years) ^a^	75.6 ± 4.4	76.0 ± 5.6	-2.854	3.754	0.276	0.784	0.091
Sex (Female/Male)^*b*^	19/1	16/2	-	-	0.487	0.485	0.11^d^
Affected side (Right/Left)^*b*^	11/9	8/10	-	-	0.422	0.516	0.11^d^
Body-mass index (kg/m^2^) ^a^	24.6 ± 3.5	24.2 ± 2.2	-2.325	1.492	-0.444	0.660	-0.143
WOMAC ^*c*^	12.9 ± 8.3	11.6 ± 8.5	-	-	-	0.588	
Muscle strength (kg)							
Hip abductors ^a^	11.7 ± 2.5	12.3 ± 2.5	-1.062	2.245	0.726	0.473	0.237
Hip adductors ^a^	9.0 ± 2.4	9.4 ± 2.3	-1.118	1.960	0.555	0.583	0.182
Hip flexors ^a^	12.1 ± 2.7	13.2 ± 3.4	-0.944	3.072	1.075	0.290	0.348
Hip extensors ^a^	12.9 ± 3.3	13.2 ± 2.9	-1.846	2.323	0.232	0.818	0.076
Knee flexors ^a^	11.8 ± 3.5	10.8 ± 3.2	-3.248	1.172	-0.953	0.347	-0.310
Knee extensors ^a^	13.7 ± 3.1	12.7 ± 3.3	-3.109	1.105	-0.965	0.341	-0.314
Functional Fitness							
30-s chair stand (no. of times) ^a^	14.4 ± 3.6	13.1 ± 3.2	-3.501	1.023	-1.111	0.274	-0.360
Functional-reach test (cm) ^a^	26.9 ± 3.7	26.7 ± 3.1	-2.553	2.040	-0.217	0.829	-0.072
10-m walk test (s) ^*c*^	10.5 ± 2.0	11.2 ± 3.9	-	-	-	0.884	-0.001

*CRE* Computer-aided rowing exercise, *CON* Control group, Data are presented as the mean ± standard deviation

^a^ *t* = independent sample t-test

^b^ *X*^*2*^ = Chi-squared test

^c^ Mann–Whitney U test

^d^ Cramer’s V

Table 2 reveals the Wilcoxon signed rank test and paired sample t-test results of the difference between the two groups in the pre- and post-tests. In the CRE group, significant differences were observed between scores in the pre- and post-tests with a small to medium effect (Cohen’s d = 0.04–0.71), including the WOMAC (*p* = 2.416 × 10^–04^), hip abductors test (*p* = 0.001), hip adductors test (*p* = 1.252 × 10^–04^), hip flexors test (*p* = 6.323 × 10^–04^), hip extensor test (*p* = 0.002), knee extensor test (*p* = 0.043), functional reach test (*p* = 0.004), and the 10-m walk test (*p* = 0.002). In the CON group, only two parameters exhibited significant changes in the pre- and post-tests, including hip abductors (*p* = 0.019) and the 10-m walk test (*p* = 0.005).

**Table 2:** Difference of pre-test and post-test WOMAC, muscle strength, and functional fitness within group.

Functional fitness parameter	CRE group (*N* = 20)			CON group (*N* = 18)		
	Mean difference (95% CI)	*p*	Cohen’ *d*	Mean difference (95% CI)	*p*	Cohen’ *d*
WOMAC ^a^		2.416*10^**–04**^	0.71		0.279	0.07
Muscle strength (kg)						
Hip abductors ^b^	-1.49(-2.25, -0.74)	**0.001**	0.13	0.99(0.19–1.79)	**0.019**	0.13
Hip adductors ^b^	-3.31(-4.76, -1.87)	1.252*10^**–04**^	0.10	-0.13(-1.04–0.77)	0.759	0.10
Hip flexors ^b^	-3.40(-4.79, -2.00)	6.323*10^**–04**^	0.18	1.13(-0.27–2.54)	0.107	0.18
Hip extensors ^b^	-0.30(-3.60, -1.00)	**0.002**	0.04	0.59(-0.34–1.51)	0.198	0.04
Knee flexors ^b^	-0.63(-1.89, 0.63)	0.307	-0.16	0.95(-0.08–1.97)	0.070	-0.16
Knee extensors ^b^	-1.01(-1.99, -0.04)	**0.043**	-0.17	0.46(-0.48–1.39)	0.317	-0.17
Functional Fitness						
30-s chair stand (no. of times) ^b^	-0.45(-2.51, 1.61)	0.653	-0.19	-0.11(-1.28–1.06)	0.843	-0.19
Functional-reach test (cm) ^b^	-3.66(-6.01, -1.31)	**0.004**	-0.04	0.21(-2.06–2.48)	0.847	-0.04
10-m walk test (s) ^a^		**0.002**	0.49		**0.005**	0.46

*CRE* Computer-aided rowing exercise, *CON* Control group, *CI* Confidence interval, bold *p* value represents reaching the significance level (*p* < .05)

^a^ Wilcoxon signed rank test was performed

^b^ paired sample t-tests were performed

Table 3 details the Quade’s ANCOVA and ANCOVA results of differences in WOMAC, muscle strength, and functional fitness between the CRE and CON groups after adjusting for age, sex, affected side, BMI, and pre-test scores. The results revealed that the WOMAC, muscle strength, and functional fitness of the participants in CRE were significantly higher than the adjusted mean scores in the post-tests than those in the CON group, including WOMAC (*p* = 0.006), hip abductors (*p* = 5.67 × 10^–5^), hip adductors (*p* = 0.001), hip flexors (*p* = 6.46 × 10^–5^), hip extensors (*p* = 4.43*10^–5^), knee flexors (*p* = 0.005), knee extensors (*p* = 0.003), and functional reach test (*p* = 0.018). The CRE intervention produced significant improvements in several indicators of WOMAC, muscle strength, and functional fitness, all of which revealed large effect sizes (Î · Â^2^ = 0.17–0.42).

**Table 3:** ANCOVA and results of the WOMAC, muscle strength, and functional fitness between the groups.

	CRE group (*N* = 20)		CON group (*N* = 18)		Adjusted post-test mean difference between groups	*F*	*p*	η^2^
	Mean	SE	Mean	SE	Mean difference (95% CI)			
WOMAC ^a^	-	-	-	-	-	8.586	0.006	0.22
Muscle strength (kg)								
Hip abductors ^b^	13.16	2.56	11.27	2.77	2.36 (1.28, 3.44)	20.98	5.67*10^–5^	0.38
Hip adductors ^b^	12.34	2.86	9.58	2.93	3.04 (1.38, 4.69)	14.03	0.001	0.31
Hip flexors ^b^	15.51	3.24	12.04	3.19	4.01(2.24, 5.78)	21.30	6.46*10^–5^	0.41
Hip extensors ^b^	15.23	2.75	12.58	2.73	2.88(1.64, 4.12)	22.54	4.43*10^–5^	0.42
Knee flexors ^b^	12.43	2.70	9.81	2.59	2.03(0.66, 3.41)	9.08	0.005	0.23
Knee extensors ^b^	14.75	2.77	12.28	2.22	1.80(0.65, 2.94)	10.28	0.003	0.25
Functional Fitness								
30-s chair stand (no. of times) ^b^	14.80	4.41	13.22	3.47	0.66(-1.96, 3.27)	0.26	0.612	0.09
Functional-reach test (cm) ^b^	30.56	4.85	26.44	4.35	3.74(0.68, 6.80)	6.20	0.018	0.17
10-m walk test (s) ^a^	-	-	-	-	-	0.68	0.417	0.02

All means, standard errors, and mean differences represent the post-test scores adjusted for age, sex, affected side, *BMI*, and pre-test scores

*CI* Confidence interval, *CRE* Computer-aided rowing exercise, *CON* Control group, bold *p* value represents reaching the significance level (*p* < .05); η^2^ = partial eta squared (η^2^) value of the ANCOVA test and Quade’s ANCOVA test.

^a^ Quade’s ANCOVA test

^b^ ANCOVA test

## Discussion

Knee OA not only results in joint pain and dysfunction of the knee joint but also leads to disabilities in daily life, economic issues, and care burden for older adults in an aging society [6]; therefore, improving the muscle weakness problem is important. Many exercise programs (conventional aerobic exercise, strength training, and cycling exercise programs) may improve muscle weakness, health conditions (pain, stiffness), and lower limb function in patients with mild knee osteoarthritis [15, 16]. However, these exercises could induce higher joint impacts and result in poor exercise adherence and intervention benefits for older patients with knee OA [51]. For example, studies have reported that land-based aerobic exercises not only positively benefit people with knee OA [52] but also induce adverse events and is not recommended for subjects with severe knee osteoarthritis because the cartilage could be damaged [53]. In addition to, there are some relative not high-impact exercise, including cycling, strenght training, water-based aerobic exercise. Therefore, to improve the effect of exercise intervention and decrease joint impact and related-induced pain in OA knees for older adults during exercise training, this study designed a CRE system that combines feedback and lower knee joint weight bearing. Compared with common strengthening exercise programs, CRE exhibits quantitative training intensity setting, game-based interaction feedback, and lower joint impacts, and is suitable for older patients with knee OA who completed all class sessions as well as facilitates exercise adherence and physical condition. Furthermore, the results revealed that the CRE can improve muscle strength and physical function in the lower limbs of older adults with mild degenerative knee OA within and between groups.

### Effect of computer-aided rowing exercises on improving the WOMAC, muscle strength, and functional fitness of older adults with knee OA

Patients with knee OA exhibit weak flexor and extensor muscle strength, which could result in poor knee joint health condition and risk of functional limitation and disabilities in daily living, economic issues, and care burden for older adults in an aging society [6, 54, 55]. In this study, after 12 weeks of CRE intervention, patients in the CRE group exhibited significant improvements in WOMAC scores, muscle strength (hip adductors, hip abductors, hip extensors, hip flexors, and knee extensors), and functional fitness (functional reach and 10-m walk tests). Compared with the CON group, the participants in the CRE group also revealed superior WOMAC, muscle strength, and balance performance after the intervention. CRE positively affected WOMAC, muscle strength, and functional fitness performance in older adults. These findings are similar to those of previous studies and revealed the short-term and long-term (2–6 months) benefits of exercise for improving pain, physical functions, and quality of life for people with knee osteoarthritis. [18, 19, 25, 28, 33, 56, 57]. However, the results revealed that the 30-s chair stand and 10-m walk tests revealed no significant improvement in the CRE group compared with the CON group. These tests focused on evaluating the functional performance requiring speed in the lower limbs [18], and the CRE approach was applied by randomizing the gaming interaction. However, in this method the movement speed in the lower limbs is not highlighted or directly trained. Furthermore, studies have revealed that pain can be induced when the knee joint is subjected to the weight-bearing condition [58]. No participant reported pain in OA knees during or after the CRE. Therefore, based on the clinical benefits of computer-aided rowing exercise for knee osteoarthritis, rowing exercise programs with lower knee joint weight-bearing conditions may be suitable for older adults with knee OA.

### Accessibility for lower joint impact exercise approaches in older adults with knee OA

The buoyancy assistance of aquatic exercise can be used to improve the muscle strength, fitness, and physical functions in patients with knee osteoarthritis [59–61]. The slow movement Tai Chi exercises also results in a low joint impact on OA knees and can be applied to improve the muscle strength and functional fitness of older patients after OA [18, 20]. However, the requirement of a large swimming pool is a major limitation of aquatic exercises. Therefore, the exercise is not accessible to knee OA patients. Infection should also be considered in older adults, especially in the COVID-19 pandemic [62]. For Tai Chi exercise, a highly experienced Tai Chi exercise instructor is required [18]. Furthermore, aquatic and Tai Chi exercises cannot set up quantitative intensity and provide individualized feedback for each patient in the exercise group, which may result in inconsistent effects [18, 24, 60]. By contrast, this study used the resistance and rowing exercise approach to establish a computer-aided feedback to the rowing exercise system. Resistance exercise is highly effective rehabilitation program for patients with knee OA in the clinic [16]. Furthermore, rowing exercises have a low impact activity with positive effects on muscle strength in people with knee OA [63–66]. Furthermore, rowing exercise is a closed kinetic chain (CKC) exercise [66]. Verma reported that CKC exercises exhibit excellent muscle strengthening and functional activities than open kinetic chain exercises in patients with osteoarthritic knees [67].

### Benefits of technology-supported feedback for people with knee OA

To provide a quantitative intensity exercise program for improving intervention effects, a computer-aided exercise system was designed to follow the resistance exercise protocols [33, 34] and set up the target force from 50% of 1-RM in the first week to 80% in the last two weeks. Target force settings were applied to trigger the interaction of the game and provide visual feedback during the exercise approach. Early studies have revealed that resistive exercise combined with visual feedback can facilitate a high muscle strength in the knee joints [68, 69]. Studies have reported that a variety of technologies can be used for visual feedback to enhance the exercise effects of individuals with knee OA [70–72]. For example, Levinger et al. used the Microsoft Kinect™ (combines high-resolution camera, infrared sensor, and providing movement correction feedback) (Microsoft Kinect™, Redmond, WA) and Nintendo Wii® balance board (uniaxial vertical force transducers and providing weight-bearing feedback) (Nintendo Co Ltd, Kyoto, Japan) and combined specific exercises (squat, sit to stand, step up, gait training, and lunges) to improve knee function during gait in people with knee osteoarthritis [73]. However, these technology-supported exercises have several limitations, including the requirement of a large space to track body movement, poor flexibility, and unchangeable exercise programs; inability to follow resistance exercise protocols to set up the exercise intensities; and inability to provide precise and individualized training programs based on the intensities to control or trigger various video games for different knee OA patients [70, 74, 75]. The LabVIEW 2015 software was used to design the program interface of the computer-aided feedback rowing exercise system flexibly and provided flexible system functions in connecting the flash games, setting the target force values in RM for interacting with flash games, data storage, and analysis to form a precision exercise approach for knee osteoarthritis patients in this study.

### Study limitations

The present study also has several limitations, including the fact that participant recruitment is constrained in many communities because of the rapid spread of COVID-19 during the study period. Therefore, several participants refused to participate in this study. Furthermore, rowing exercises require the trunk, upper limbs, and lower extremities to function simultaneously [76, 77]. Although we collected the leg press forces of both lower limbs to play video game in CRE group, we found that all participants grasp two rowing handles to maintain their stability in the upper body during the computer-aided rowing exercise program. Thus, the stability in the upper body may play important role in performing rowing exercise program in older people. However, we did not measure and analyze the support force to maintain upper body stability, which may reveal different stability requirements for upper limb force support when performing rowing exercises in elderly adults; this can be considered in future studies.

## Conclusions

This study revealed that computer-aided feedback rowing exercise with lower joint impacts positively affected knee joint health, muscle strength, and physical function in older adults with mild knee OA. The findings and feedback of the participants in this study revealed that computer-aided feedback rowing exercise is an appropriate approach for providing individualized and precise exercise programs for older adults with knee OA.

## Data Availability

All the data used in this study are available from the corresponding author upon reasonable request.

## Abbreviations

OA: Osteoarthritis
ADLs: Activities of daily living
CRE: Computer-aided rowing exercise
SPSS: Statistical package of the social sciences
ANCOVA: Analysis of covariance
WOMAC: Western Ontario and McMaster Universities osteoarthritis index
